# Phylogenetic Distribution and Diversity of Bacterial Pseudo-Orthocaspases Underline Their Putative Role in Photosynthesis

**DOI:** 10.3389/fpls.2019.00293

**Published:** 2019-03-14

**Authors:** Marina Klemenčič, Johannes Asplund-Samuelsson, Marko Dolinar, Christiane Funk

**Affiliations:** ^1^Department of Chemistry, Umeå University, Umeå, Sweden; ^2^Department of Chemistry and Biochemistry, Faculty of Chemistry and Chemical Technology, University of Ljubljana, Ljubljana, Slovenia; ^3^Science for Life Laboratory, School of Engineering Sciences in Chemistry, Biotechnology and Health, KTH Royal Institute of Technology, Solna, Sweden

**Keywords:** orthocaspase, metacaspase, photosynthesis, pseudo-enzyme, cyanobacteria

## Abstract

Orthocaspases are prokaryotic caspase homologs – proteases, which cleave their substrates after positively charged residues using a conserved histidine – cysteine (HC) dyad situated in a catalytic p20 domain. However, in orthocaspases pseudo-variants have been identified, which instead of the catalytic HC residues contain tyrosine and serine, respectively. The presence and distribution of these presumably proteolytically inactive p20-containing enzymes has until now escaped attention. We have performed a detailed analysis of orthocaspases in all available prokaryotic genomes, focusing on pseudo-orthocaspases. Surprisingly we identified type I metacaspase homologs in filamentous cyanobacteria. While genes encoding pseudo-orthocaspases seem to be absent in Archaea, our results show conservation of these genes in organisms performing either anoxygenic photosynthesis (orders Rhizobiales, Rhodobacterales, and Rhodospirillales in Alphaproteobacteria) or oxygenic photosynthesis (all sequenced cyanobacteria, except *Gloeobacter*, *Prochlorococcus*, and *Cyanobium*). Contrary to earlier reports, we were able to detect pseudo-orthocaspases in all sequenced strains of the unicellular cyanobacteria *Synechococcus* and *Synechocystis. In silico* comparisons of the primary as well as tertiary structures of pseudo-p20 domains with their presumably proteolytically active homologs suggest that differences in their amino acid sequences have no influence on the overall structures. Mutations therefore affect most likely only the proteolytic activity. Our data provide an insight into diversification of pseudo-orthocaspases in Prokaryotes, their taxa-specific distribution, and allow suggestions on their taxa-specific function.

## Introduction

**FIGURE 1:**
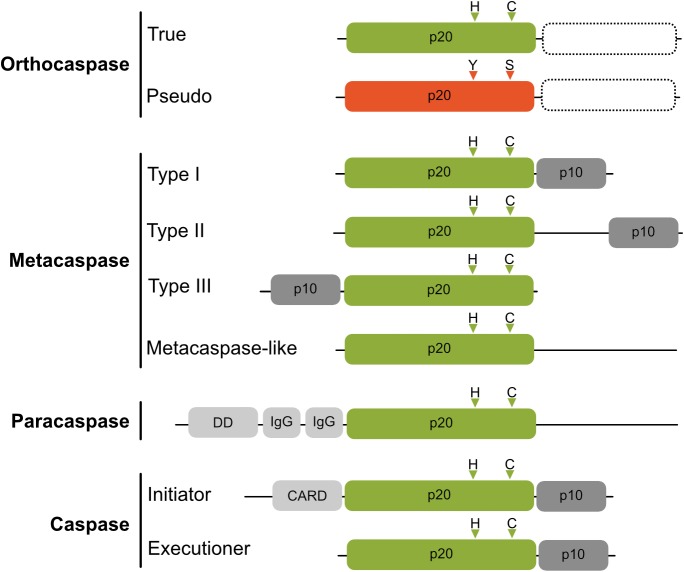
Schematic domain organization of proteins belonging to C14 cysteine proteases. The p20-like domain is colored in green with positions of catalytic His and Cys residues shown as triangles above the domains. Pseudo-orthocaspases are colored in orange, with the most common active site substitution (His to Tyr and Cys to Ser) shown also as orange triangles. True- as well as pseudo-orthocaspases can, but not necessarily do, contain additional domains on the C-terminal (shown as rectangle with a dashed border). The regulatory p10 domain is shown in dark gray and additional domains in light gray (IgG, immunoglobulin-like domain; DD, death domain; CARD, caspase activation and recruitment domain). Figure is not drawn to scale.

Metazoan caspases (cysteine-aspartic proteases), playing essential roles in programmed cell death, are synthesized as inactive zymogens, which for activation are cleaved into a catalytic, large (p20) and regulatory, small (p10) subunit (Earnshaw et al., 1999). The family of proteins containing the p20 fold, however, is much broader and contains beside the archetypic caspase proteases (subclass C14A, reviewed by Shalini et al., 2015) also metacaspases (Minina et al., 2017), paracaspases (Jaworski and Thome, 2016) and orthocaspases (Klemenčič and Funk, 2018a), classified as C14B and found in diverse organisms ranging from plants over slime molds and fungi to bacteria (Figure 1). Both subclasses contain active proteases, whose active sites contain catalytic histidine and cysteine residues (HC dyad). Unlike caspases, which cleave their substrates after negatively charged aspartic acid residues, members of the C14B family specifically degrade substrates with basic amino acid residues at position P1 (Vercammen et al., 2004; Hachmann et al., 2012; Klemenčič et al., 2015). Metacaspases represent the largest sub-family of these caspase-homologs and depending on their domain structure they are subdivided into different types (Vercammen et al., 2004). Type I metacaspases contain a proline-rich repeat and a zinc-finger motif in the N-terminal prodomain (Vercammen et al., 2004), whereas type II metacaspases lack these motifs and are hallmarked by the presence of an extended linker region between the p20-like and p10-like domains. Recently, genes encoding a third type of metacaspases (type III metacaspases) have been identified in algae that arose from secondary endosymbiosis (Choi and Berges, 2013), those enzymes harbor the p10 domain N-terminal to the p20 domain. While metacaspases are found in plants, fungi and protists, paracaspases are more similar to caspases and were detected in animals and slime molds. They are classified by lack of a p10 domain, but contain immunoglobulin-like (Ig) domains (Uren et al., 2000).

Since the identification of caspases as the key enzymes in programmed cell death of animals, similar functions were sought for metacaspases of Planta and Protozoa. Although initial reports were focused on portraying direct involvement of metacaspases in processes of programmed cell death (Bozhkov et al., 2005; He et al., 2008; Minina et al., 2013), roles of metacaspases were soon shown to exceed mere functions of cell execution and to function in multiple non-death roles (Lee et al., 2008). Additionally, some protozoan type I metacaspases were discovered to lack the complete catalytic dyad rendering them proteolytically inactive; of the five metacaspases encoded in the genome of *Trypanosoma brucei*, TbMC1 and TbMC4 contain HS and YS substitutions (Szallies et al., 2002). Enzymes lacking functional amino acids in their active sites are very common. They are collectively termed pseudo-enzymes (Eyers and Murphy, 2016) and sometimes also referred to as non-enzymes, dead enzymes, prozymes or ‘zombie’ proteins (Murphy et al., 2017). These proteins are structurally homologous to their active relatives, but lack catalytic activity due to mutations of critical amino acid residues. Even though they are believed to be catalytically inert, increasing data reveal them being functional, important proteins. Currently best studied and most intensively analyzed are pseudo-kinases (reviewed by Byrne et al., 2017) and pseudo-phosphatases (Reiterer et al., 2014), as well as pseudo-proteases, which are gaining the interest of researchers. Pseudo-variants of orthocaspases were already observed in the first genome-wide analysis of Cyanobacteria (Jiang et al., 2010), and later 16% of all identified prokaryotic p20-containing sequences were found to contain substitutions in the catalytic dyad with YS being the most common one (Asplund-Samuelsson et al., 2012). Interestingly, in the majority of unicellular Cyanobacteria the only orthocaspase encoded in the genome is the pseudo-variant (Klemenčič and Funk, 2018a).

To better understand the distribution of pseudo-orthocaspases, we performed a comprehensive *in silico* analysis on all prokaryotic caspase homologs with focus on variants, which instead of the conserved HC dyad harbor other amino acid residues. We show that these pseudo-enzymes are especially abundant in organisms performing oxygenic and to a lower extent in organisms executing anoxygenic photosynthesis (Cyanobacteria and Alphaproteobacteria, respectively). Their possible function in prokaryotic organisms is discussed.

## Materials and Methods

### Identification of Caspase Homologs

Caspase-homologs including orthocaspases were identified using a p20 domain profile hidden Markov model (HMM) constructed using *hmmbuild* from HMMER v3.1b2^1^ and the first 457 columns of the Pfam Peptidase_C14 (PF00656) seed alignment, downloaded from https://pfam.xfam.org/. The p20 HMM was used in an *hmmsearch* versus UniProt release 2018_10 (134,066,004 sequences), downloaded from https://www.uniprot.org/ on 12 November 2018. Hits with a best one domain *E*-value lower than 0.0001 were accepted. Further filtration was then performed by alignment to the p20 HMM using *hmmalign*, followed by removal of sequences with a gap in the His or Cys catalytic dyad positions. Finally, sequences with a p20 domain length not falling within a range of two standard deviations from the mean of the remaining sequences, i.e., 101–200 amino acids, were also removed. For identification of p20-containing proteins in organisms of genera *Synechocystis, Synechococcus*, *Prochlorococcus*, and *Cyanobium* we performed a DELTA-BLAST protein homology search^2^ using the previously identified *Synechocystis* sp. PCC 6803 p20 domain (Jiang et al., 2010) as a query.

### Classification of Caspase Homologs

The accepted caspase homologs from UniProt were subjected to detailed classification of the catalytic dyad, domain architecture, and taxonomy. The alignment to the p20 HMM was used to extract the amino acid residues corresponding to the His and Cys catalytic dyad positions for each sequence. Next, an *hmmsearch* with four additional HMMs was performed versus the sequence dataset, aiming to identify metacaspases and paracaspases via domain architecture. The p10 domain was identified using a p10 HMM constructed with *hmmbuild* from the last 211 columns of the Pfam Peptidase_C14 seed alignment. Paracaspases were identified by having at least one Pfam Immunoglobulin (Ig) (PF00047), Ig_2 (PF13895), or Ig_3 (PF13927) domain beginning its alignment before the start of the p20 alignment. We applied domain cutoff scores of 30.0 for p10, 21.8 for Ig, 27.0 for Ig_2, and 30.0 for Ig_3. Since a few proteins had multiple p20 domains, a cutoff score was implemented for those as well. First, the maximal p20 score in each sequence was extracted, then the cutoff was set to the minimal value among those maxima, i.e., 35.2. The p20 and p10 domain alignment positions were compiled into a domain architecture. Sequences with a p10 domain starting after the start of the p20 domain were classified as type I metacaspases if the distance between the domains was 66 amino acids or shorter, and otherwise as type II metacaspases. The 66 amino acid limit was based on known interdomain distances (Choi and Berges, 2013) and constitutes the mid-point between the upper limit of a 95% confidence interval (mean plus two standard deviations) for type I metacaspase interdomain distances, and the lower limit of a 95% confidence interval (mean minus two standard deviations) for type II metacaspase interdomain distances. Sequences with a p10 domain ending before the start of a p20 domain were classified as type III metacaspases. If there were more than one p20 or p10 domain, the sequence was classified as ambiguous. Furthermore, the p10 domains were aligned to the p10 HMM using *hmmalign* and the amino acid residues in the conserved Cys and Asp positions (Choi and Berges, 2013; Klemenčič and Funk, 2018b) were extracted to further classify the metacaspases. Finally, each sequence in the dataset was matched to taxonomic data using their NCBI taxonomy IDs and the NCBI taxonomy database, downloaded from https://www.ncbi.nlm.nih.gov/ on 4 October 2018.

### Identifying Protein Sequences Motifs

Protein sequence motifs in addition to the p20 domain were identified by submitting FASTA protein sequences to the MOTIF web page^3^, which performs a search with a protein query sequence against PROSITE, NCBI-CDD and Pfam motif libraries.

### Phylogenetic Tree Construction

In order to trace, visualize, and explore the sequence diversity and evolution, we performed sequence alignments of the p20 domains and phylogenetic tree construction. The p20 domains were extracted from archaeal and bacterial caspase homologs, as well as three metazoan caspases serving as an outgroup (human caspase-3 and caspase-8, and *Caenorhabditis elegans* cell death protein 3; UniProt sequence IDs P42574, Q14790, and P42573), using *hmmalign* versus the p20 HMM with the *--trim* option, followed by gap removal with seqmagick v0.6.2^4^. The p20 domains were aligned using MAFFT v7.271 (Katoh and Standley, 2013), and FastTreeMP v2.1.8 SSE3 (Price et al., 2010) was used for phylogenetic tree construction. Alignment and tree construction was performed for three sequence subsets: All archaeal and bacterial p20 domains, the subset that contained a mutation in the His – Cys catalytic dyad (pseudo-variants), and cyanobacterial p20 domains. Identity of each sequence to each other and to the outgroup sequences was calculated for each of the three alignments as the fraction of matching positions, including internal gaps, unless present in both sequences being compared, and excluding positions with gaps at the ends of the sequences. The phylogenetic trees were visualized using the Interactive Tree of Life (iTOL) v4.1 online service at https://itol.embl.de/ (Letunic and Bork, 2016). To emphasize highly supported clades, branches with a bootstrap support lower than 0.85 were deleted using iTOL.

### Sequence Logo Generation

Based on the substitution in the His – Cys catalytic dyad, we recognized two distinct cyanobacterial clades in the phylogenetic tree of mutant p20 domains: YN and YS, labeled by the most prominent catalytic dyad substitutions (see Supplementary Table S1). The p20 domains from each clade as well as all cyanobacterial p20 domains with an active dyad (HC) were aligned per sequence group using MAFFT v7.271. The alignments were then transformed to HMMs with *hmmbuild* and submitted to Skylign^5^ in order to generate sequence logos based on above background information content, thus illustrating the features in each group of sequences.

## Results

### Prokaryotes Contain a Rich Pool of Caspase Homologs

**FIGURE 2:**
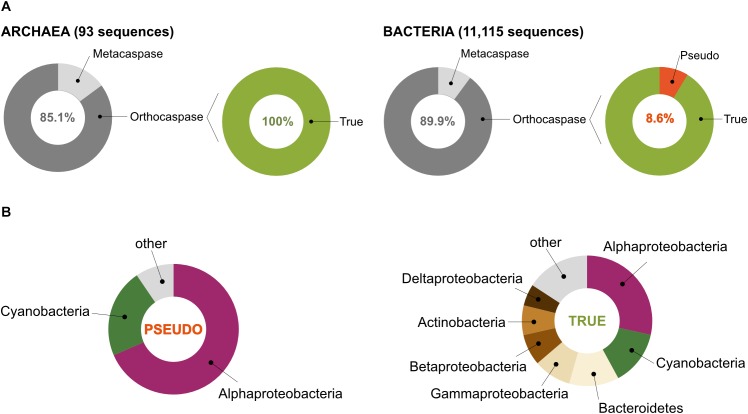
Prokaryotes contain metacaspases and orthocaspases. **(A)** Our study identified 11,208 sequences with homology to the p20 domain. Among these, 93 were found in organisms belonging to Archaea and 11,115 to Bacteria. Sequences homologous to the p10 domain could be identified in 11 archaeal and 1,121 bacterial sequences (metacaspases), the rest represent orthocaspases. Of the bacterial orthocaspases, 8.6% lacked the conserved His – Cys dyad and were termed pseudo-orthocaspases. No pseudo-metacaspases or pseudo-orthocaspases were identified in archaeal organisms. **(B)** Of all pseudo-variants detected, the majority was found in Alphaproteobacteria or Cyanobacteria (left panel), while the true-variants (presumably active enzymes) are distributed among the bacterial groups (phylum; class for Proteobacteria) ranging from Alphaproteobacteria to Deltaproteobacteria (right panel). Only groups containing containing more than 5% of sequences encoding meta- and orthocaspases were named in the chart.

According to the MEROPS database of proteolytic enzymes^6^, family C14 comprises proteins that contain a characteristic p20 domain with a highly conserved caspase/hemoglobinase fold. This domain accommodates two catalytically crucial residues: a histidine and a cysteine residue (His – Cys, HC), situated on the surface of the domain (Uren et al., 2000; McLuskey and Mottram, 2015). Performing an HMM-search in the most recent UniProt database, which contains 2,936,402 archaeal and 94,326,796 bacterial sequences, representing 4,042 and 94,934 organisms (based on unique taxonomy IDs), respectively, we identified 11,208 protein sequences with homology to the p20 domain in prokaryotic organisms (Supplementary Table S1). Among those, only 93 belonged to Archaea, while the rest (11,115) represented bacterial caspase homologs (Figure 2A).

In order to distinguish between the structural subtypes of caspase homologs (meta-, para-, orthocaspases), we first tried to identify paracaspases by searching for the presence of immunoglobulin-like (Ig) domains within the identified sequences. Neither Archaea nor Bacteria contained sequences encoding paracaspases, confirming their absence in prokaryotes. The presence of a p10 domain, located either N- or C- terminal to the p20 domain, defines metacaspases. Sequences with high similarity to the p10 domain could be identified in 11 archaeal and 1,121 bacterial sequences, representing 14.9% and 10.1% of all p20-containing sequences, respectively (Figure 2A). No type III metacaspases were detected in either of the two prokaryotic superkingdoms. While archaeal sequences were found to contain only type I metacaspases, 10 bacterial metacaspases contained a linker region longer than 66 amino acid residues between the p10 and p20 domain, which would classify them as type II metacaspases. These sequences represented less than 1% of all metacaspase sequences and were not analyzed further. We concluded that bacterial metacaspases generally can be classified as metacaspases of type I.

All remaining sequences lacking the p10 domain were termed orthocaspases. Similar to bacterial orthocaspases (Jiang et al., 2010; Asplund-Samuelsson et al., 2012), also archaeal ones were found to contain additional domains on their N- and C-termini. Among the most prevalent were a C-terminal FGE-sulfatase domain (found in 8 proteins), an EF-hand motif (found in 7 proteins) and an N-terminal Big_7 motif, which is a bacterial Ig-like domain (found in 15 proteins).

### Pseudo-Orthocaspases Are Present in Bacteria, but Absent in Archaea

The presence of the HC dyad within the p20 domain is an important prerequisite for proteolytic activity. Analysis of all archaeal meta- as well as orthocaspases revealed the presence of this conserved functional dyad in all 93 sequences (Figure 2A). However, some bacterial sequences lacked this catalytic site: 4 out of 1,121 metacaspases and 858 out of 9,994 orthocaspases contained substitutions of either one or both catalytic residues. To distinguish between the presumably active enzymes with conserved catalytic dyad and the presumably inactive ones with substituted/missing active sites, we denoted the sequences “true-” or “pseudo-”, respectively, in analogy to the newly established nomenclature for catalytically deficient enzymes (Eyers and Murphy, 2016) (Figure 2A).

**FIGURE 3:**
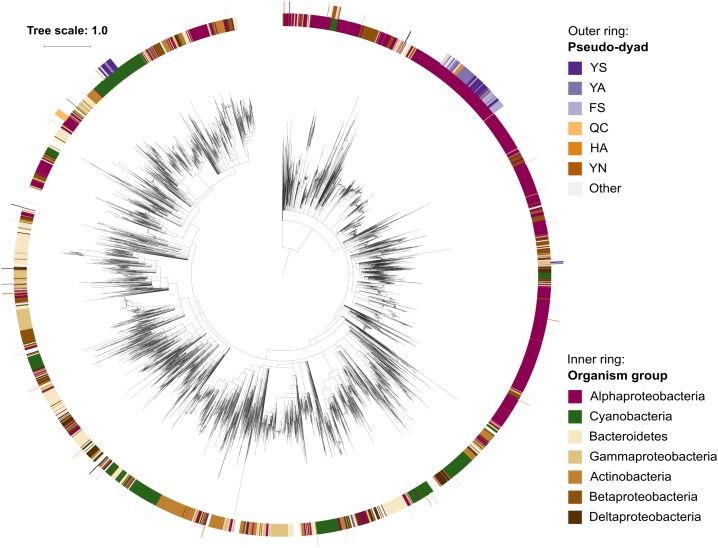
Phylogenetic relationships among prokaryotic caspase homologs. The tree is based on the p20 domain of 11,208 bacterial and archaeal caspase homologs identified in this study, and three metazoan caspases (UniProt IDs P42574, Q14790, and P42573), included as outgroup. The inner ring displays the most abundant organism groups (phylum; class for Proteobacteria), while the outer ring displays the presence and type of substitutions in the catalytic dyad found in pseudo-orthocaspases. Branches with bootstrap support below 0.85 have been deleted. The scale bar shows substitutions per site. The alignment consisted of 2,077 positions and the mean identity to the outgroup sequences was 9.6% (s.d. 2.0%), while 19.0% (s.d. 7.7%) among the prokaryotic sequences. The alignment and a Newick tree are supplied in Supplementary Data Sheet S1.

Since pseudo-metacaspases represented only 0.36% of all bacterial sequences with homology to the p20 domain, they were statistically irrelevant for further analysis. Bacterial pseudo-orthocaspases, however, represented a significant fraction of 8.6% of all bacterial caspase homologs, warranting further analysis. While 90% of all pseudo-orthocaspases were found in organisms belonging to either Alphaproteobacteria (582 sequences) or Cyanobacteria (193 sequences) (Figure 2B), only few pseudo-variants were identified in other phyla (e.g.,10 out of 1078 sequences in Bacteroidetes, 3 out of 636 sequences in Actinobacteria, and 2 out of 667 sequences in Betaproteobacteria). Notably, this uneven distribution did not reflect the distribution of true-orthocaspases in Bacteria (Figure 2B, right panel), where 28.4% of all identified sequences belong to Alphaproteobacteria, and 13.6% to Cyanobacteria, followed by Bacteroidetes (12.4%), Gammaproteobacteria (9.3%), Betaproteobacteria (7.7%), Actinobacteria (7.4%), and Deltaproteobacteria (5.4%). Pseudo-orthocaspases in Alphaproteobacteria and Cyanobacteria therefore seem to have diversified during evolution. To gain a comprehensive overview of the phylogenetic relationships between all prokaryotic caspase homologs, we constructed a phylogenetic tree based on the alignment of their p20 sequences (Figure 3). The tree revealed great sequence diversity and a highly mixed contribution from various taxa within subclades, which could be explained by paralogs of potentially diverse function, or extensive horizontal gene transfer. Note, however, that a more detailed analysis is required to attempt to measure the contribution of processes such as horizontal gene transfer. Alphaproteobacterial sequences are placed close to the root, i.e., metazoan caspases, which is in line with the proposed alphaproteobacterial origin of caspases (Aravind and Koonin, 2002). Pseudo-orthocaspases were mainly found clustered in three basal clades and two more distant clades. The presence of such clades suggests conserved functions of those pseudo-orthocaspase variants. Their distribution among true-variants might indicate multiple independent emergences.

### Pseudo-Orthocaspases Are a Distinctive Trait of Alphaproteobacteria Performing Anoxygenic Photosynthesis

**FIGURE 4:**
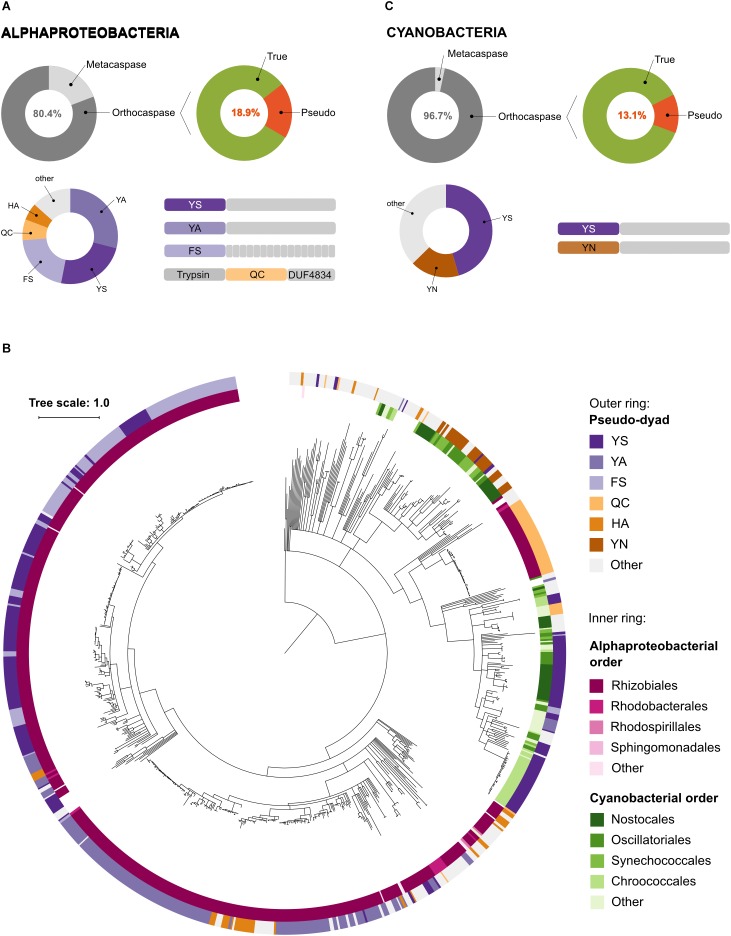
Pseudo-orthocaspases are abundant in Alphaproteobacteria and Cyanobacteria. **(A)** In Alphaproteobacteria we identified 3,826 caspase homologs: 751 metacaspases and 3,075 orthocaspases. 582 enzymes lacked the conserved His – Cys dyad and were termed pseudo-orthocaspases. The most common substitutions of the catalytic dyad and their C-terminal domain organizations are shown in the lower panel. Pseudo-enzymes containing FS substitutions consistently had tetratricopeptide repeats C-terminally to the p20 domain (drawn as small gray rectangles). Trypsin denotes the trypsin-like domain, while DUF4843 denotes a well-conserved fold without known function. **(B)** Phylogenetic relationships among bacterial pseudo-orthocaspases. The tree is based on the p20 domain of all 862 bacterial pseudo-orthocaspases identified in this study, and three metazoan caspases (UniProt IDs P42574, Q14790, and P42573), included as the out-group. The inner ring displays Alphaproteobacterial and Cyanobacterial taxonomic orders, while the outer ring displays the amino acids found to substitute the catalytic dyad. Branches with bootstrap support below 0.85 have been deleted. The scale bar shows substitutions per site. The alignment consisted of 360 positions and the mean identity to the outgroup sequences was 11.3% (s.d. 2.5%), while 29.9% (s.d. 18.3%) among the bacterial sequences. The alignment and a Newick tree are supplied in Supplementary Data Sheet S1. **(C)** In Cyanobacteria we identified 1,525 caspase homologs: 50 metacaspases and 1,475 orthocaspases. 193 enzymes lacked the conserved His – Cys dyad and were termed pseudo-orthocaspases. The most common substitutions of the catalytic dyad and their C-terminal domain organizations are shown in the lower panel.

Although Alphaproteobacteria were found to be rich in metacaspases, 80.4% of alphaproeobacterial sequences containing a p20 domain lacked the p10 domain and were classified as orthocaspases (Figure 4A). Among these, 18.9% contained a substitution in the HC dyad, classifying them as pseudo-orthocaspases. Phylogenetically these pseudo-variants seem to be limited to Rhizobiales, Rhodobacterales and Rhodospirillales, the three orders of anaerobic phototrophic purple bacteria. Notably, more than 96% of these pseudo-orthocaspases belonged to organisms of Rhizobiales (561 out of 582), 14 were Rhodobacterial and only two belonged to organisms of Rhodospirillales (Figure 4B). Although true-orthocaspases were found to be abundant also in photosynthetic Betaproteobacteria (orders Burkholderiales and Rhodocyclales) and Gammaproteobacteria (order Chromatiales), these organisms lacked pseudo-variants; neither were pseudo-orthocaspases detected in green-sulfur bacteria (Chlorobi). In green-nonsulfur bacteria (Chloroflexi), three sequences corresponding to pseudo-orthocaspases were found. Two of them were found in the class Ktedonobacteria (species *Ktedonobacter racemifer* DSM 44963 and *Ktedonobacter* sp.), while the third pseudo-orthocaspase was found in the class Anaerolineae (strain *Anaerolineaceae* bacterium 4572_78). Both classes belong to non-phototrophic Chloroflexi.

More detailed analysis of the substitutions of the HC dyad in pseudo-orthocaspases of Rhizobiales showed the most common dyads to be Tyr – Ser (YS), Tyr – Ala (YA) or Phe – Ser (FS). While often both amino acid residues were substituted, also Gln – Cys (QC) and His – Ala (HA) combinations seem to be common. We further observed that each of the substitution variants corresponded to a certain overall protein domain architecture (Figure 4A). While p20 domains with YS or YA substitutions contained mostly prolonged C-termini without identifiable domains, those with a FS dyad consistently also contained tetratricopeptide repeats (TPR), which are known scaffolds mediating various protein – protein interactions (Blatch and Lässle, 1999). Moreover, proteins in which the p20 domain contained a QC dyad almost exclusively also contained an N-terminal trypsin-like domain. Examination of this trypsin-like domain identified all three conserved amino acid residues of the catalytic triad of serine proteases (His – Asp – Ser, HDS), suggesting proteolytic activity. At the C-terminus of these proteins a domain of unknown function (DUF4384) was identified, which often co-occurs in proteins with N-terminal kinase domains. This alphaproteobacterial, QC-dyad containing clade seems to be phylogenetically different to other alphaproteobacterial pseudo-orthocaspases, which cluster together in close proximity to the root (Figure 4B). Instead, the QC variants appear to be closer related to distal cyanobacterial pseudo-orthocaspase clades.

### Cyanobacteria Contain a Rich Pool of Pseudo-Orthocaspases

Although cyanobacteria were thought not to contain metacaspases (Choi and Berges, 2013), we identified 50 protein sequences, which classify as type I metacaspases (Figure 4C). All of them belong to genera of filamentous cyanobacteria: *Anabaena*, *Calothrix*, *Coleofasciculus*, *Lyngbya*, *Moorea*, and *Nostoc.* However, as only few species within these genera contained metacaspases, metacaspases most likely have been acquired via horizontal gene transfer during the evolution of these complex cyanobacteria.

More than 96% of all cyanobacterial p20-containing proteins were found to be orthocaspases. The diversification of true-orthocaspases has been investigated previously (Jiang et al., 2010; Asplund-Samuelsson et al., 2012) and is thus not discussed here. However, 13% of all cyanobacterial orthocaspases were found to lack a functional HC dyad. Unlike in Alphaproteobacteria, where proteins containing pseudo-p20 domains were found to be order-specific, analysis of cyanobacterial genomes revealed the presence of at least one pseudo-orthocaspase in each organism of this phylum, ranging from simple cyanobacteria, such as *Microcystis*, unicellular nitrogen-fixing *Gloeocapsa*, to filamentous nitrogen-fixing *Nostocales.* Their catalytic dyad was most often substituted by YS and no other domains were identified (Figure 4C). In rare cases, the catalytic dyad was replaced by YA, FS, or YY. Only in the evolutionary old cyanobacterial genus *Gloeobacter*, i.e., *Gloeobacter violaceus* PCC 7421 and *Gloeobacter kilaueensis* JS1, only true orthocaspases could be found. Additionally, organisms belonging two genera of marine picocyanobacteria were found to lack any p20-containing proteins; *Prochlorococcus*, a simple and heavily streamlined organism (Partensky and Garczarek, 2009) and *Cyanobium*.

### Synechococcus and Synechocystis Strains Carry p20-Containing Proteins

Small unicellular organisms, especially strains of freshwater *Synechocystis* and marine *Synechococcus*, have previously been reported to lack orthocaspases (Jiang et al., 2010), and indeed also in our list of p20-domain containing cyanobacteria these organisms seemed to be absent. However, one pseudo-orthocaspase has previously been identified in *Synechocystis* sp. PCC 6803 (Asplund-Samuelsson et al., 2012). To circumvent the rather strict constraints for identification of the p20 domain in our automated search, we performed additional manual analyses within the NCBI database. Proteins with homologous sequences to the p20 domain of *Synechocystis* 6803 were identified in both genera and are listed in Table 1; the alignment of their p20 domains is shown in Supplementary Figure S1. Surprisingly, each organism identified was found to contain a single gene encoding an orthocaspase and all these p20-domains harbored single- or double substitutions in the HC dyad. YS substitutions were the most common motifs in *Synechococcus* and YG in *Synechocystis*. All identified sequences therefore represent pseudo-orthocaspases.

**Table 1:** List of manually identified proteins with a p20 domain in the genera of *Synechococcus* and *Synechocystis*.

	NCBI ID	His – Cys conservation	True- or Pseudo-variant
***Synechococcus***			
PCC 7502	WP_015167715.1	His – Gly	Pseudo
PCC 7003	WP_065712853.1	Tyr – Ser	Pseudo
PCC 8807	WP_065715717.1	Tyr – Ser	Pseudo
PCC 7002	WP_012305740.1	Tyr – Ser	Pseudo
NKBG042902	WP_030006746.1	Tyr – Ser	Pseudo
BDU130192	WP_099239871.1	Tyr – Ser	Pseudo
PCC 73109	WP_062431693.1	Tyr – Ser	Pseudo
PCC 7117	WP_065709860.1	Tyr – Ser	Pseudo
JA-2-3B_a(2-13)	WP_011432265.1	Tyr – Gly	Pseudo
PCC 7336	WP_017327236.1	Tyr – Ser	Pseudo
NIES-970	WP_096415465.1	Tyr – Ser	Pseudo
NKBG15041c	WP_081699644.1	Tyr – Ser	Pseudo
PCC 6312	WP_015123192.1	Tyr – Gly	Pseudo
PCC 7335	WP_006457254.1	His – Gly	Pseudo
***Synechocystis***			
PCC 6803	WP_010873177.1	Tyr – Gly	Pseudo
PCC 6714	WP_028947383.1	Tyr – Gly	Pseudo
PCC 7509	WP_009633488.1	Tyr – Gly	Pseudo

### Only Nitrogen-Fixing Multicellular Cyanobacteria Contain Two Distinct Pseudo-Orthocaspases

**FIGURE 5:**
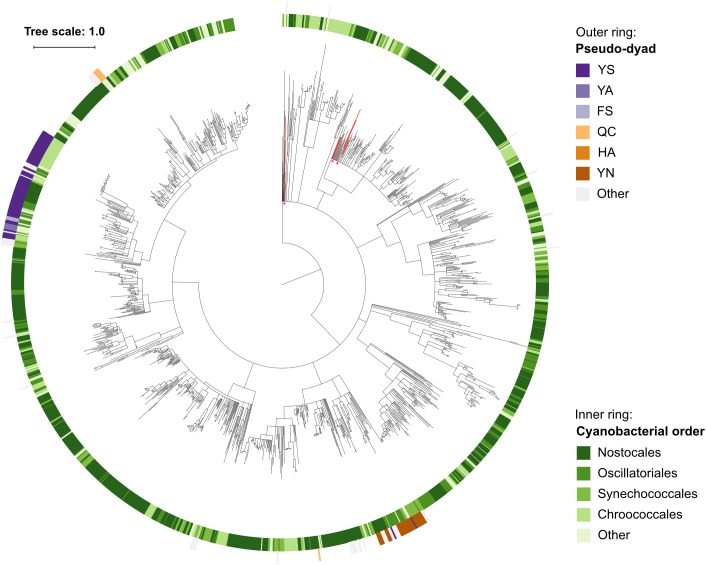
Phylogenetic relationship among Cyanobacterial caspase homologs. The tree is based on the p20 domain of all 1,525 Cyanobacterial caspase homologs identified in this study, and three metazoan caspases (UniProt IDs P42574, Q14790, and P42573) included as the outgroup. The inner ring displays taxonomic orders, while the outer ring displays the presence and catalytic-dyad substitution of pseudo-orthocaspases. The six sequences originating from genus *Gloeobacter* are emphasized in red and with asterisks. Branches with bootstrap support below 0.85 have been deleted. The scale bar shows substitutions per site. The alignment consisted of 668 positions and the mean identity to the outgroup sequences was 9.8% (s.d. 1.8%), while 21.9% (s.d. 11.0%) among the Cyanobacterial sequences. The alignment and a Newick tree are supplied in Supplementary Data Sheet S1.

Within the set of cyanobacterial pseudo-orthocaspases a large group of sequences contained YS and YN pseudo-catalytic dyads (Figure 4C) and lacked any identifiable domain at the C-terminus of the p20-bearing polypeptide chain. We observed that these YS or YN containing p20 domains clustered in two distinct clades, while proteins with other substitutions were scattered throughout the phylogenetic groups (Figure 5). Moreover, p20 domains with an YN motif could only be identified in multicellular nitrogen-fixing Cyanobacteria; examples of such genera are: *Leptolyngbya*, *Calothrix*, *Microcoleus*, *Nostoc* as well as *Planktothrix*. As opposed to metacaspases, which were (as previously mentioned) detected only in some filamentous cyanobacterial species, pseudo-orthocaspases with an YN dyad could be found in all analyzed organisms of these genera. It should, however, be noted that YN variants were found to be absent in unicellular nitrogen-fixing Cyanobacteria (e.g., genera: *Cyanothece*, *Gloeocapsa*) as well as filamentous non-heterocystous Cyanobacteria (e.g., *Prochlorothrix hollandica* PCC 9006), thus suggesting specific function of the YN variant in organisms with both properties: multicellularity and nitrogen-fixation. Only multicellular nitrogen-fixing cyanobacteria therefore contain two distinct pseudo-orthocaspase types, one with an YN substitution and the other with an YS substitution.

### Acidic Nature of the Specificity Pocket Is Conserved in Cyanobacterial Pseudo-Orthocaspases

**FIGURE 6:**
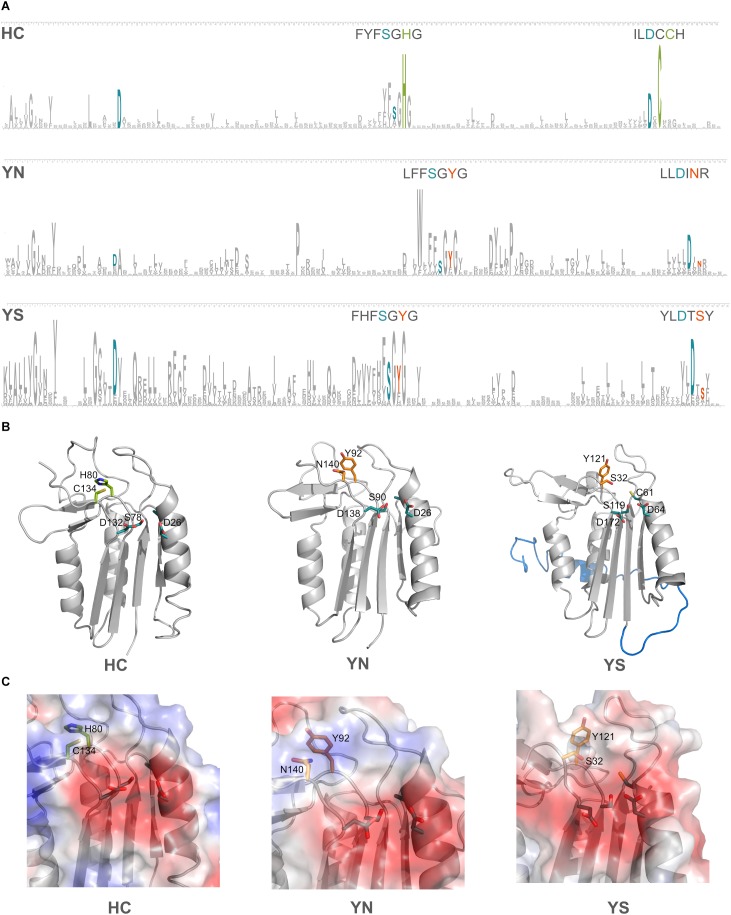
Substitutions in the catalytic dyad of cyanobacterial pseudo-orthocaspases influence the overall folds of neither the p20 domain nor the specificity pocket. **(A)** Sequence logos for positions in the p20 domain are shown for the true- (HC) and two pseudo-variants (YS and YN) in Cyanobacteria. See Methods section “Sequence Logo Generation” for details and Supplementary Table S1 for the corresponding sequences (121 for YS and 49 for YN). The height of a certain amino acid residue corresponds to its conservation (above background information content). Amino acid residues of the active site are colored according to the proteolytic functionality (H and C in green and Y, S, and N in orange). In cyan, residues known to be involved in formation of the specificity pocket are shown. For better visibility of the motifs surrounding the catalytic residues, these stretches are written out above logos. **(B)** Models for each of the three variants are shown in ribbon representation. The p20 domains of three *Nostoc* sp. T09 proteins: HC, A0A252E3G8; YS, A0A252D9J9; YN, A0A252EC77 were submitted to the I-TASSER Suite (https://zhanglab.ccmb.med.umich.edu/I-TASSER/) (Yang et al., 2015) and protein structures were visualized using PyMOL (DeLano Scientific; http://www.pymol.org). All three domains are drawn as ribbons with active site residues and the key amino acid residues involved in substrate coordination as sticks. The N-terminal region, resembling a TAT-signal peptide, is shown in blue in the YS variant. **(C)** Surface potentials of all three specificity pockets are shown in magnified view; blue indicates basic amino acids, red acidic amino acids. Side chains of the amino acids in the catalytic dyad and specificity pocket are shown as sticks.

Domains C-terminal to the p20 domain, which consistently accompanied certain HC-substitutions have been mentioned earlier. We now investigated if additional amino acid changes in the p20 domain occurred in the pseudo-enzymes. Four amino acid residues have been shown to be crucial for substrate coordination in the p20-domain of type I metacaspase (TbMC2) of *Trypanosoma brucei*: Cys92, Asp95, Ser159 and Asp211 (McLuskey et al., 2012); the two negatively charged amino acid residues were shown to be highly important in accommodating basic residues of substrates. In the p20 domain sequences of prokaryotic pseudo-orthocaspases, these two Asp residues seem to be highly conserved, independent of the substitutions occurring within the catalytic dyad. Sequence logos were constructed for the two most common cyanobacterial substitutions of the HC-dyad, YS and YN, and compared to all cyanobacterial true-variants (Figure 6A). Highest conservation was found among those amino acid residues forming the specificity pocket and surrounding the catalytic site, while other residues forming the p20 domain not only differ between pseudo- and true-variants, but also are relatively diverse within each variant. Differences in primary protein structure, however, were not reflected in the tertiary folding (Figure 6B), representative models of the p20 domains of three orthocaspases from *Nostoc sp.* T09 (Supplementary Figure S2) contain similar overall folds.

The two well-conserved Asp residues (Figure 6A) are located within the substrate binding pocket in the models (Figure 6C). Not only do the pseudo-variants contain a well-conserved fold, they can accommodate substrates with basic charge, despite being presumably inactive proteases. Notably different is an extended N-terminal domain of approximately 30 amino acid residues found in the YS variant. In the model this extension forms an alpha-helix (marked in blue color, located behind the p20 domain in Figure 6B). This extension seems to be conserved in all identified Synechococcal orthocaspases containing a YS substitution (Supplementary Figure S1) and therefore might have a specific function.

## Discussion

The caspase-hemoglobinase is one of the most conserved folds, present in proteins of prokaryotic as well as eukaryotic organisms. It is composed of four beta-sheets surrounded by three alpha-helices that form a compact globular domain (Aravind and Koonin, 2002). This fold is the key domain and common denominator of the members of the caspase superfamily, including caspases, metacaspases, paracaspases and orthocaspases. Prokaryotic caspase homologs were first generally termed metacaspases (Asplund-Samuelsson et al., 2012), however, due to the lack of the p10 domain they were renamed to metacaspase-like proteases. Doubts concerning their proteolytic activity (Choi and Berges, 2013) were dispelled, when MaOC1, a cyanobacterial p10-lacking member was shown to be proteolytically active and even to exhibit the highest catalytic efficiency among non-metazoan caspase homologs (Klemenčič et al., 2015). Due to their evolutionary importance and structural as well as functional distinctiveness, these prokaryotic metacaspase-like proteins were termed orthocaspases.

In this study we were able to identify the presence of not only orthocaspases, but also metacaspases in prokaryotes. Although metacaspases were earlier reported to be rather scarce in Alphaproteobacteria and absent in Cyanobacteria (Choi and Berges, 2013), we could identify p10 domain containing enzymes in both groups. With the exception of few metacaspases in Bacteroidetes, all archaeal as well as bacterial metacaspases contain a conserved Asp residue at the N-terminus of the p10 domain (as evident in Supplementary Table S1), which was recently shown to be crucial for calcium-binding and activity in eukaryotic type III metacaspases (Klemenčič and Funk, 2018b). A common proteolytic mechanism therefore might be conserved in bacterial and eukaryotic metacaspases.

Additionally, we were able to identify pseudo-proteases among the caspase-homologs, which seem to be restricted to prokaryotic orthocaspases. Substitutions of both catalytic residues were most commonly observed in these pseudo-orthocaspases, similar to the pseudo-metacaspase of *Trypanosoma brucei* TbMC4, in which YS replaces the catalytic dyad (Szallies et al., 2002). This YS variant is also most prevalent among bacterial pseudo-orthocaspases. Beside these substitutions in the active site, the overall folds of the p20-domain or the substrate binding pocket seem not to differ between true- and pseudo-variants as denoted by modeling of cyanobacterial true- and pseudo-variants (Figure 6). No protein structures have unfortunately been resolved yet, neither for ortho- nor pseudo-orthocaspases, therefore we cannot exclude larger conformational shifts or the presence of additional amino acid residues, which would sterically hinder substrate binding. In SMIPP-Ss, for example, a multigene family of house dust mite allergen group 3 homologs, which are classified as members of the S1-like family (Wilson et al., 2003), a conserved tyrosine residue at position 200 is predicted to block substrate access to the active site (Fischer et al., 2009). A highly conserved tryptophan residue also can be found in YN pseudo-orthocaspases, located only a few amino acid residues in front of the “catalytic” tyrosine (Figure 6A). In our model this residue is predicted to be part of a beta-sheet and positioned adjacent to one of the alpha helices (Supplementary Figure S3) and could therefore not directly interfere with coordination of the substrate.

The most striking difference of YS pseudo-orthocaspases compared to the presumably active orthocaspases is the presence of an additional N-terminal domain. Motif prediction software was not able to assign this domain despite its N-terminal double RR and C-terminal A-x-A sequences, specifying cleavage by a signal peptidase (Supplementary Figures S1, S2). These two features are characteristic for a TAT (Twin-Arginine Translocation) signal sequence, which in Cyanobacteria translocates proteins that are fully folded either into the periplasm or the thylakoid lumen (Frain et al., 2016). We hypothesize that cyanobacterial YS-pseudo-orthocaspases are involved in photosynthetic processes and/or thylakoid membrane biogenesis in Cyanobacteria, as (i) these enzymes are present in all cyanobacteria except *Gloeobacter* (which lack thylakoid membranes), *Prochlorococcus* and *Cyanobium* and (ii) in *Synechocystis sp.* PCC 6803 as well as in *Microcystis aeruginosa* PCC 7806 genes encoding these pseudo-orthocaspases are strongly light-regulated (Straub et al., 2011; Saha et al., 2016). Further, we hypothesize that the picocyanobacteria of genera *Prochlorococcus* and *Cyanobium* lost their orthocaspases altogether while adapting to the sparse marine environment, possibly gaining a similarly simplified and pseudo-orthocaspase independent physiology as that of *Gloeobacter*.

Within Alphaproteobacteria, only (anoxygenic) photosynthetic organisms carry pseudo-variants, an observation further supporting our hypothesis that pseudo-orthocaspases are involved in photosynthetic processes in Prokaryotes. However, pseudo-orthocaspases are absent in green- and purple-sulfur bacteria and only three pseudo-orthocaspase sequences within green non-sulfur photosynthetic bacteria (including the phylum Chloroflexi) were detected. The photosynthetic reaction centers (RC) of anoxygenic photosynthetic prokaryotes have a common origin, but diverged in the course of evolution (e.g., Cardona, 2016). RCII of Alphaproteobacteria and Chloroflexi has evolved from the same ancestral RCII, which in oxygenic photosynthetic Cyanobacteria gave rise to Photosystem II. Although this seems to link functions of pseudo-orthocaspases to RCII, absence of pseudo-orthocaspases in phototrophic Chloroflexi and some Cyanobacteria as well as in RCII-bearing Gemmatimonadetes (Zeng et al., 2014) hampers this interpretation.

Pseudo-orthocaspases are presumably proteolytically inactive and based on our data we assume that they evolved from their active relatives: (i) Various pseudo-variants are scattered throughout phylogenetically loosely related organisms (Figure 3), suggesting the emergence of pseudo-enzymes to be an example of convergent evolution. Such spontaneous and independent appearance in diverse species is highly reminiscent of the distribution of phototrophy across the bacterial tree, with phototrophic clades dispersed across the tree rather than forming a single clade (Fischer et al., 2016; Ward et al., 2018). (ii) A combination so specific as the HC dyad can be found throughout evolution in all clades (the distant metazoan caspases still retain the catalytic HC dyad throughout millions of years of separated evolution). (iii) Within cyanobacteria, where pseudo-orthocaspases are highly common, the earliest-branching cyanobacterium, *Gloeobacter*, contains only the true variant, appearing to have diverged before the emergence of the distal YS pseudo-orthocaspases (Figure 5). Enzymes, which have lost their proteolytic function in comparison to their true-variants have been observed to function as dynamic scaffolds involved in signaling cascades, or can act as competitors, scavenging substrates, which are released under certain circumstances (Reynolds and Fischer, 2015). The pseudo-metacaspase of *T. brucei*, TbMC4, has been shown to be crucial for blood-stage parasite cytokinesis and virulence during mammalian infection. Since it is processed by a true-metacaspase, TbMC3, it is part of the catalytic cascade, thus indirectly regulating the function of its active counterparts (Proto et al., 2011). The pseudo-p20 cFLIP_L_ variant (cellular FLICE-like inhibitory protein long form), an inactive caspase-8 homolog in humans, has been shown to act as dimerization partner of caspase-8 (Yu et al., 2009). While these well-studied pseudo-variants act in conjunction with their true-relatives, small unicellular Cyanobacteria harbor only a pseudo-variant, suggesting these proteins to have functions independent of active counterparts. Most likely also prokaryotic pseudo-orthocaspases found in organisms, which additionally harbor true-orthocaspases, are not involved in the regulation of their proteolytic activity. It should be noted that genes encoding cyanobacterial pseudo-orthocaspases, especially the YS variant, are highly expressed: metatranscriptomic data from the cyanobacterium *Nodularia spumigena* show consistent and relatively high expression levels in correlation to other genes, suggesting even a “house-keeping” function of the corresponding proteins (Asplund-Samuelsson et al., 2016).

The high conservation of pseudo-orthocaspases in Cyanobacteria and their putative involvement in processes of oxygenic photosynthesis on one hand, with their absence in eukaryotic Chlorophyta and Streptophyta (Supplementary Table S1) on the other hand raise many questions regarding their function. Although some functions involving pseudo-caspase homologs have been described for Protista and Metazoa, the loss of pseudo-orthocaspases within the cyanobacterium-plastid transition is intriguing. One also should bear in mind that the presence of the catalytic dyad might not necessarily correspond to proteolytic activity: the role of the type I metacaspase AtMC2 from *Arabidopsis thaliana* was found to be independent of the presence of putative catalytic residues (Coll et al., 2010). Further *in vitro* as well as *in vivo* characterizations are necessary for our understanding of the function of the p20 domain in the various cellular environments they occur: from a simple unicellular bacterium to humans.

## Data Availability

All datasets generated for this study are included in the manuscript and/or the Supplementary Files.

## Author Contributions

All authors designed the study. MK and JA-S analyzed the data and drafted the manuscript. All authors revised the manuscript.

## Conflict of Interest Statement

The authors declare that the research was conducted in the absence of any commercial or financial relationships that could be construed as a potential conflict of interest.

## Footnotes

1 http://hmmer.org/

2 https://blast.ncbi.nlm.nih.gov/

3 https://www.genome.jp/tools/motif/

4 https://fhcrc.github.io/seqmagick/

5 http://skylign.org/

6 https://www.ebi.ac.uk/merops/
