# Supplementary material for: Phylogenetic Distribution and Diversity of Bacterial Pseudo-Orthocaspases Underline Their Putative Role in Photosynthesis
Source: Front Plant Sci. 2019 Mar 14;10:293. doi: 10.3389/fpls.2019.00293 (PMC6426788; doi:10.3389/fpls.2019.00293)
Supplement: Supplementary file 1 [file Table_1.DOCX]

Supplementary Material

# Supplementary Data and Tables

## Supplementary Table 1

Supplementary Table 1 is available separately and contains information on all caspase homologs detected in the UniProt 2018_10 database:

Table 1.XLSX

## Supplementary Data Sheet 1

Supplementary Data Sheet 1 is available separately and contains alignments of prokaryotic orthocaspases and the resulting phylogenetic tree files in Newick format:

Data Sheet 1.ZIP

# Supplementary Figures

## Supplementary Figure 1


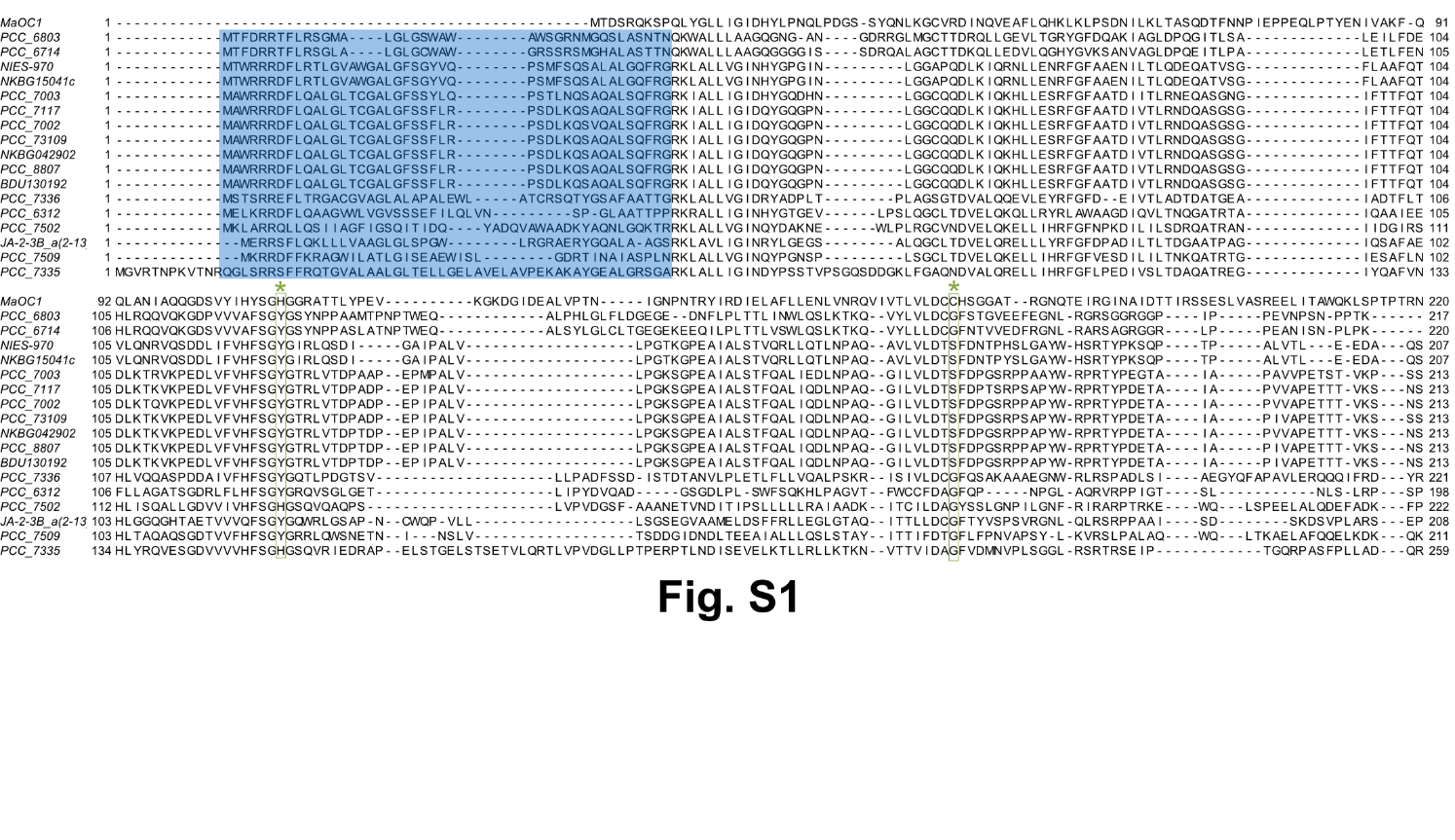


**Fig. S1: Alignment of the pseudo-orthocaspase p20 domains of genera *Synechocystis* and *Synechococcus* in comparison to the true-orthocaspase MaOC1** **(Klemenčič, Novinec, and Dolinar 2015).** Positions of the HC catalytic dyad are marked with a green rectangle and an asterisk. In blue, amino acid sequences forming a TAT-signal peptide are shown. NCBI IDs of all sequences are listed in Table 1. Sequence alignment was performed using PROMALS and the figure was generated using Jalview.

## Supplementary Figure 2


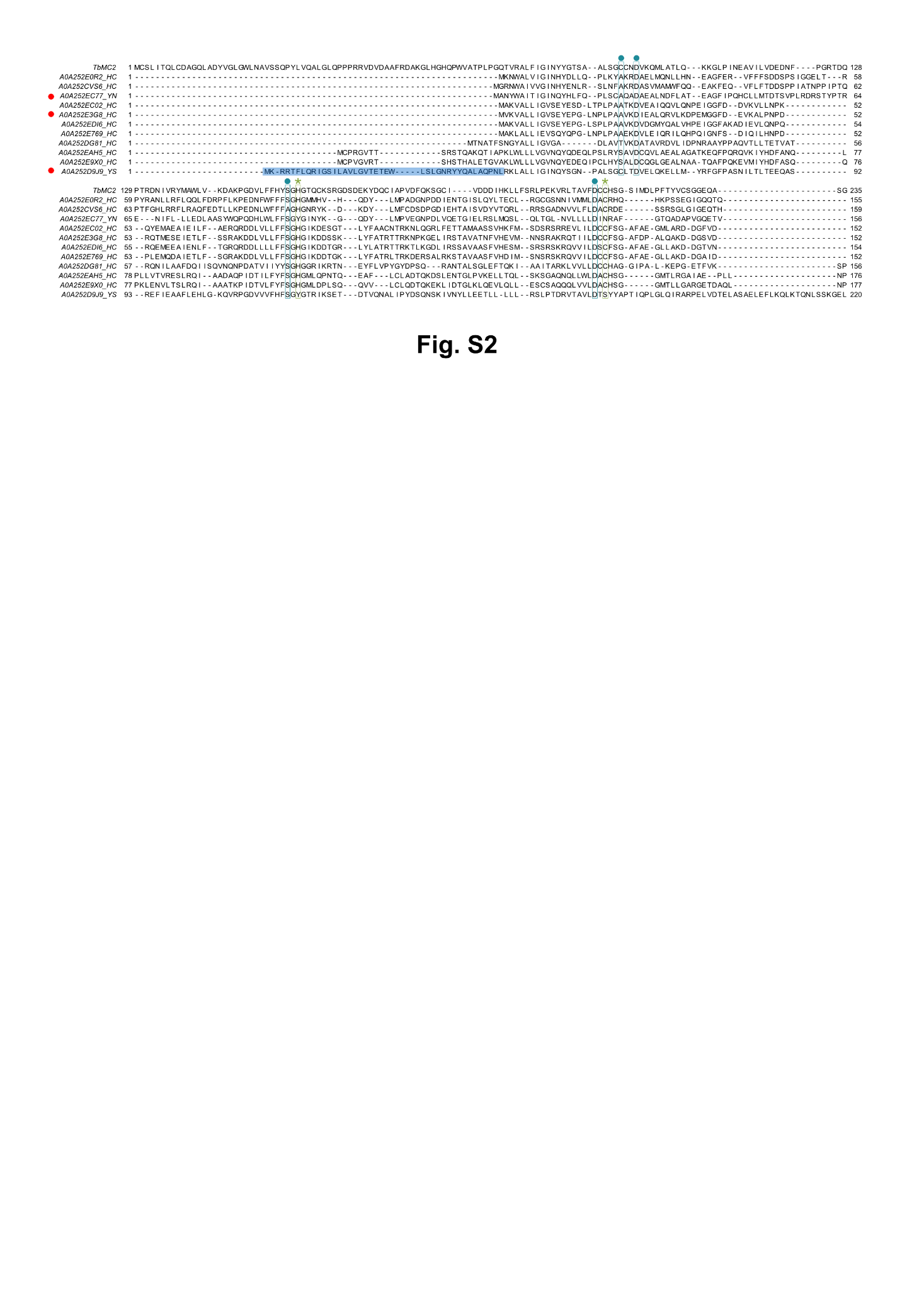


**Fig. S2: Alignment of all p20 domains of orthocaspases from the Cyanobacterium *Nostoc* sp. T09.** IDs correspond to UniProt IDs in Table S1; HC, YS or YN denote amino acid residues of their active sites. Sequences that were used for domain modeling, are denoted with red dots. Positions of the HC catalytic dyad are marked with a green rectangle and an asterisk. In blue, an amino acid sequence forming a TAT-signal peptide is shown. Sequence alignment was performed using PROMALS and the figure was generated using Jalview.

## Supplementary Figure 3


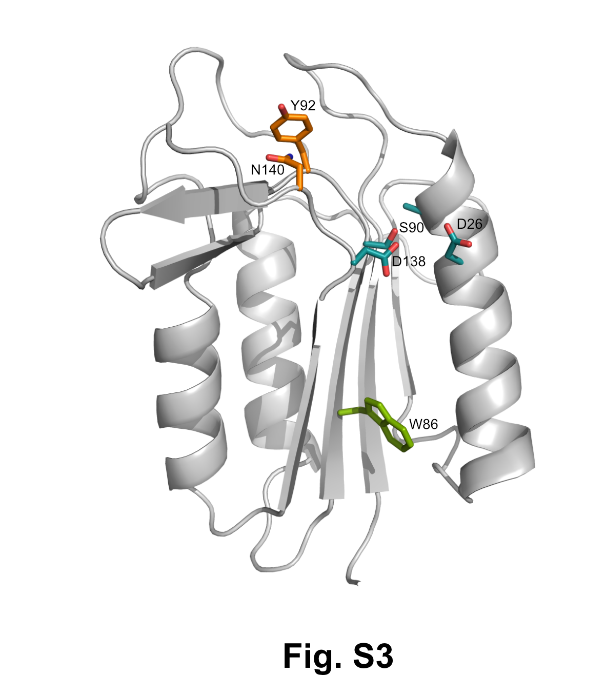


**Fig. S3: Model of the p20 domain of a pseudo-orthocaspase variant YN from *Nostoc* sp. T09**. The amino acid sequence of the protein with UniProt ID A0A252EC77 was submitted to the I-TASSER Suite (<https://zhanglab.ccmb.med.umich.edu/I-TASSER/>) (Yang et al. 2015) and protein structure was visualized using PyMOL (DeLano Scientific; [http://www.pymol.org](http://www.pymol.org/)). The domain is drawn as ribbon with active site residues and the key amino acid residues involved in substrate coordination as sticks. A very conserved tryptophan residue is also shown in green sticks representation.
